# Incidental Detection of Preputial Calculus in a Patient with Partial Phimosis: Is it as Rare as We Believed?

**DOI:** 10.15388/Amed.2021.28.1.10

**Published:** 2021-03-09

**Authors:** Evangelos N. Symeonidis, Chrysovalantis Toutziaris, Antonios Katsimantas, Georgios Dimitriadis

**Affiliations:** 1^st^ Department of Urology, Aristotle University of Thessaloniki, School of Medicine, “G. Gennimatas” General Hospital, Thessaloniki, Greece; 1^st^ Department of Urology, Aristotle University of Thessaloniki, School of Medicine, “G. Gennimatas” General Hospital, Thessaloniki, Greece; Department of Urology, Mediterraneo Hospital, Glyfada, Athens, Greece; 1^st^ Department of Urology, Aristotle University of Thessaloniki, School of Medicine, “G. Gennimatas” General Hospital, Thessaloniki, Greece

**Keywords:** Preputial, Calculi, Phimosis, Urolithiasis, Poor Hygiene, Circumcision

## Abstract

**Summary. Background:**

Preputial stone disease is the rarest type of urolithiasis. Adult males with severe phimosis and poor hygiene are mainly affected.

**Case Presentation:**

A 90-year-old male sought treatment for steadily worsening urinary frequency, intermittency, incontinence, and pain at the tip of his penis of 3-days duration. Clinical examination revealed a palpable distended urinary bladder, a partial phimosis and a round, hard on palpation, and partly ulcerative lesion at the tip of the foreskin. A single, 1 cm in maximum diameter stone, was incidentally discovered beneath the prepuce and subsequently removed from the preputial sac. The patient refused further treatment with circumcision, and opted for conservative therapy of benign prostate hyperplasia.

**Conclusion:**

Personal hygiene remains the cornerstone in the prevention of the preputial calculi formation, while circumcision represents the mainstay of treatment for definite stone removal and elimination of the precipitating causes.

## Abstract (lt)

**Santrauka. Įvadas:**

Apyvarpės akmenligė yra rečiausias urolitiazės tipas. Dažniausiai šia liga serga suaugę vyrai, turintys sunkią fimozės formą ir netinkamai besilaikantys intymios higienos.

**Klinikinis atvejis:**

90-metis vyriškis kreipėsi į gydytojus dėl padažnėjusio šlapinimosi, nutrūkstančios šlapimo srovės, šlapimo nelaikymo ir tris dienas trunkančio varpos galvutės skausmo. Atlikti medicininiai tyrimai parodė akivaizdžiai padidėjusią šlapimo pūslę, dalinę fimozę ir apvalią, apčiuopiamai sukietėjusią ir iš dalies išopėjusią žaizdelę ant apyvarpės galiuko. Po apyvarpe atsitiktinai buvo aptiktas vienas, maždaug 1 cm skersmens akmuo, kuris vėliau iš apyvarpės maišelio buvo pašalintas. Pacientas atsisakė cirkumcizijos ir pasirinko konservatyvų gėrybinės prostatos hiperplazijos gydymą.

**Išvada:**

Asmens higiena yra pagrindinis apyvarpės akmenų susidarymo prevencijos būdas, o cirkumcizija – pagrindinis gydymo metodas, kurį naudojant efektyviai šalinami akmenys ir jų atsiradimo priežastys.

***Reikšminiai žodžiai:*******apyvarpė, akmenys, fimozė, urolitiazė, prasta higiena, cirkumcizija

## Introduction

Preputial stone disease (PSD) remains an exceptionally under-reported type of urolithiasis since its first presentation by Robert Clarke in 1794 [1]. It occurs mainly in adult males and less often in children with coexistent urologic or neurologic diseases [2]. The primary causal factor leading to the development of preputial stones is severe phimosis [1]. Herein, we present an interesting case of PSD in an adult patient with phimosis, while at the same time underlining the importance of personal hygiene in the prevention of PSD formation. To the best of our knowledge, this is the fourth report of PSD in Europe.

## Case Presentation

A 90-year-old male of low socio-economic status presented in the emergency department with acute urinary retention and a 3-day-long history of lower urinary tract symptoms (LUTS). More specifically, he complained of steadily worsening urinary frequency, intermittency, post-micturition dribbling, urge incontinence, and constant pain at the tip of his penis. His past medical history was significant for acute myocardial infarction, diabetes mellitus type 2, hypertension, dyslipidemia, and mild dementia.

**Figure. 1:**
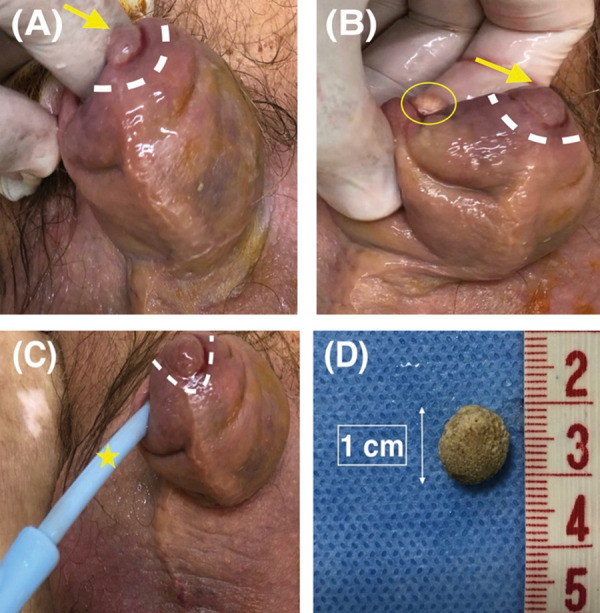
**(A)** Photo demonstrating the round partly ulcerative lesion (yellow arrow, white dotted line) on the left side of the tip of the foreskin. Note the thickened, irritated and soaked prepuce. **(B) **Photo demonstrating stone’s removal. The index finger was inserted in the preputial sac through the phimotic ring (yellow circle). The skin lesion gradually receded following stone’s removal (yellow arrow, white dotted line). **(C) **Urinary catheter insertion to relieve acute urinary retention (yellow asterisk). The round partly ulcerative lesion is also depicted (white dotted line) **(D) **The preputial stone, measuring 1 cm in maximum diameter.

Clinical examination revealed a nontender, palpable distended urinary bladder. The foreskin appeared swollen and was painful on examination, and a round, about 1 cm in diameter, ulcerative lesion was noted on the left side of the tip of the foreskin. Intermittent, spontaneous leakage of small amounts of urine could be easily noticed through the tight preputial opening. We tried to retract the foreskin in order to identify the external urethral meatus and proceed with catheterization. After insertion of the index finger in the preputial cavity, tight adhesions were felt and a single, oval stone impacted beneath the foreskin was seen. A 1 cm in maximum diameter yellow-gray colored nonobstructing stone was eventually removed and the external urethral meatus was identified *(Figure 1)*. Despite the stone’s firm embedment underneath the prepuce, the urethral meatus appeared intact hypothesizing that benign prostate hyperplasia could have triggered LUTS’s exacerbation. Moreover, no congenital urethral abnormalities could be detected.

Subsequently, a 20-Fr Foley catheter was inserted and 1100 ml of urine was drained. Serum creatinine was mildly elevated (1.9 mg/dL, normal values 0.6–1.2 mg/dL) while blood chemistry and urinalysis were within normal limits. Abdominal ultrasonography showed mild bilateral dilatation of the upper urinary tract. Plain X-ray of the kidney, ureter, and bladder did not reveal any radiopaque shadows. 

During hospitalization, the patient was managed conservatively with hydration, antibiotics, and topical application of antifungal cream. The urine culture obtained during catheterization was negative. Following an uneventful recovery, he was discharged on the second-day post-admission and listed for circumcision. Nevertheless, he refused surgical treatment and opted only for the treatment of LUTS with alpha-blockers. 

## Discussion

There is a paucity of critical data in the existing body of literature pertinent to PSD [1]. Previously published reports derive from underdeveloped countries, especially from India [3]. In European literature, there is scarce evidence for this specific type of urolithiasis [4-6]. To the best of our knowledge, our report is the fourth PSD in Europe. 

Similarly, to our case, in 1997, Sonnex et al. described the presence of three preputial calculi consisting of smegma in a 24-year old patient with a partially retractable foreskin and coexistent balanoposthitis [6]. The number and size of stones usually vary, while there is a predominance in uncircumcised adults or elders with severe phimosis [1,2,7]. Less commonly, the condition may appear in uncircumcised children with coexistent urologic or neurologic diseases [2]. Low socioeconomic status and poor hygiene are secondary risk factors, as confirmed in our case [4].

Approaching the pathogenesis of PSD, we recognize three different types of preputial calculi: (a) those originating from inspissated smegma with lime salts, (b) calculi originating from precipitation of urinary salts because of urinary retention in the preputial cavity, which may be accompanied by an infection usually caused by urea-splitting bacteria, and (c) migratory calculi from the upper urinary tract to the preputial sac [1,5,7]. Regarding their composition, preputial stones consist of inspissated smegma, smegma and urinary salts or urinary salts alone [3].

Smegma in the subpreputial space may act as a nidus promoter for stone formation while further inducing local inflammation, adhesion formation, and preputial stenosis with subsequent obstruction [1,6].

In our case, smegma solidification was possibly incriminated as the phimosis was not severe enough to cause urinary stasis, salt precipitation, or entrapment of a migratory stone. Moreover, this process could explain the reason behind local infection and adhesions that were encountered between the glans penis and inner prepuce in our patient. Besides, there was no indication of lithiasis elsewhere in the urinary tract, and the urine culture was sterile. 

Common presenting symptoms range from voiding difficulties, foul-smelling penile discharge, and chronic balanoposthitis to penile pain and acute urinary retention [2,5,7,8,9]. Neglected preputial stones can also lead to fistula formation in the preputial skin and even more seriously in penile cancer [4,8].

Physical examination usually suffices as calculi can be easily palpated in the preputial sac. At the same time, imaging modalities such as ultrasound and X-ray of the kidney, ureter, and bladder support diagnostically by unveiling lithiasis in the rest of the urinary tract [1,4]. 

Considering the above, dorsal slit incision or circumcision represent the surgical approaches for definitive treatment, thus permitting stone removal and elimination of the precipitating cause [1,4,5].

## Conclusion

In conclusion, we emphasized the importance of personal hygiene in the prevention of preputial stone formation. Prompt recognition, circumcision, and weaning from causative agents are needed to approach this infrequent urological entity. Physicians should maintain a high index of clinical suspicion, thus performing a diligent genitourinary examination of the external male genitalia.

## References

[ref1] Palinrungi MA, Kholis K, Syahrir S, Syarif, Faruk M. Multiple preputial stones: A case report and literature review. Int J Surg Case Rep. 2020;70:87-92. doi: 10.1016/j.ijscr.2020.04.041. 10.1016/j.ijscr.2020.04.041 PMC7229234 32416489

[ref2] Chong TH, Asyraf MZ, Hayati F, Azizan N, Sahid NA, Ting JRS, Zakaria AD. Giant Preputial Calculus: The First Reported Case in Malaysia. Case Rep Surg. 2018 9 18;2018:4606259. doi: 10.1155/2018/4606259. 30319829 10.1155/2018/4606259 PMC6167587

[ref3] Ellis DJ, Siegel AL, Elder JS, Duckett JW. Preputial calculus: a case report. J Urol. 1986 8;136(2):464-5. doi: 10.1016/s0022-5347(17)44910-x. 3525859 10.1016/s0022-5347(17)44910-x

[ref4] Tuğlu D, Yuvanç E, Yılmaz E, Batislam E, Gürer YK. Unknown complication of preputial calculi: preputial skin fistula. Int Urol Nephrol. 2013 10;45(5):1253-5. doi: 10.1007/s11255-013-0496-x. 23884725 10.1007/s11255-013-0496-x

[ref5] Spataru RI, Iozsa DA, Ivanov M. Preputial calculus in a neurologically-impaired child. Indian Pediatr. 2015 2;52(2):149-50. doi: 10.1007/s13312-015-0591-4. 25691187 10.1007/s13312-015-0591-4

[ref6] Sonnex C, Croucher PE, Dockerty WG. Balanoposthitis associated with the presence of subpreputial “smegma stones”. Genitourin Med. 1997 12;73(6):567. doi: 10.1136/sti.73.6.567. 9582487 10.1136/sti.73.6.567 PMC1195951

[ref7] Kekre G, Kothari P, Gupta A, Patil P, Kamble RS, Dikshit K, Deshpande A. A rare case of preputial calculi in a child with balanitis xerotica obliterans: A short communication. *African Journal of Urology*. 2016 22(3):227-229. doi: 10.1016/j.afju.2015.05.002

[ref8] Mohapatra TP, Kumar S. Concurrent preputial calculi and penile carcinoma--a rare association. Postgrad Med J. 1989 4;65(762):256-7. doi:10.1136/pgmj.65.762.256. 2594604 10.1136/pgmj.65.762.256 PMC2429261

[ref9] Yuasa T, Kageyama S, Yoshiki T, Okada Y. Preputial calculi: a case report. Hinyokika Kiyo. 2001 7;47(7):513-5. 11523140

